# Remembering the order of serially presented objects: A matter of time?

**DOI:** 10.1177/2398212819883088

**Published:** 2019-10-23

**Authors:** G.R.I. Barker, O. Evuarherhe, E.C. Warburton

**Affiliations:** School of Physiology, Pharmacology and Neuroscience, University of Bristol, Bristol, UK

**Keywords:** Order memory, trace decay, recognition memory, rat

## Abstract

Remembering the sequence, in which stimuli are encountered or events have occurred, is a key process in episodic memory and can also facilitate recognition memory. Rodents, when presented with a sequence of objects, will explore the object encountered first; yet, whether this behaviour is because the rodents spontaneously encode the order of stimuli presentation or because of relative familiarity or temporal decay is unknown. Here, we tested sequence memory in rats using a series of spontaneous preference tasks. Experiment 1 demonstrated that when rats are presented with a sequence of four objects, with an inter-sample interval of 5 min or 1 h, they preferentially explored the object presented earlier in the list irrespective of the inter-sample interval. We then demonstrated that such memory for order was not affected by increasing or decreasing the inter-sample interval between the middle objects (Experiment 2). Finally, we showed that memory for order is not a function of absolute object familiarity, as animals showed clear discrimination between the objects presented in the sample phases and a novel object, independent of the sample objects’ position in the sequence (Experiment 3). These results show that animals are able to encode the order of objects presented in a sequence, and as such temporal order memory is not achieved using the process of relative or absolute familiarity or temporal decay.

## Introduction

In humans, episodic memory, that is, the memory for unique personal experiences, is rich in sequence information as any episodic experience includes a series of events occurring in a particular order (Tulving, 1983). Judgements of prior occurrence of familiar stimuli can also be made using order information, and such discriminations can, therefore, also facilitate recognition memory (Fozard and Weinert, 1972; Hannesson et al., 2004a, 2004b; Mitchell and Laiacona, 1998). The ability to remember a sequence and discriminate the order of items in the sequence has been demonstrated in non-human primates (Chen et al., 1997; Devine et al., 1979; Templer and Hampton, 2013; Wright et al., 1985) and rodents (Bolhuis and Van Kampen, 1988; Fortin et al., 2002; Kesner et al., 2002; Roberts et al., 2008) as well as in humans, and across species it has been argued that the formation and retrieval of sequences involves multiple cognitive processes (for review, see Marshuetz and Smith, 2006). One process proposed is that of trace decay (Staddon and Higa, 1999). In other words, as memory of a stimulus decays over time, a stimulus presented earlier in the sequence will have a weaker memory trace than an item presented subsequently, thus animals could use differences in the strength of the memories to discriminate the order of stimuli presentation. Thus, increasing the delay between stimuli to be discriminated will increase the relative difference in memory trace strength and should enhance discrimination of the order of presentation.

The purpose of the present experiments was to test this trace-decay hypothesis of sequence memory. In these experiments, rats were exposed to a sequence of four objects in four sample phases (S1–S4), with a fixed interval between each sample phase. In the test phase, the rats were presented simultaneously with two objects from different sample phases, and the memory was tested using a spontaneous object exploration task. According to the decay hypothesis, the object presented earlier in the list will be represented by a weaker memory trace than that from later in the list. Thus, the earlier item will appear less familiar than the later item, and more exploratory behaviour should be directed towards the earlier item. A natural prediction that follows from this hypothesis is that the preference for exploring the earlier item will be stronger when the test stimuli consist of the first object from S1 and the last object from S4 than when it consists of the second object from S2 and the third object from S3. This prediction was tested in Experiment 1 with an interval between presentations of successive items of either 5 min or 1 h. The trace-decay hypothesis was tested further in Experiment 2, where the interval between S2 and S3 was either a short (10 min) or a long (160 min) interval, while the interval between S1 and S4 was held constant. The decay hypothesis predicts that rats will more easily differentiate between objects from S2 and S3 with the long rather than the short interval. In Experiment 3, we examined the animals’ ability to discriminate between the items presented in S1–S4 and a novel item, thus examining object familiarity discrimination to test whether the memory traces of the test objects were equivalent. Finally, additional Bayesian analyses were conducted to examine the probability of the null hypothesis (*H*_0_) being true.

## Methods

### Subjects

All experiments were conducted in male Lister Hooded rats (weighing 200–250 g at the start of the experiments; Harlan, Hull, UK). The animals were housed, in pairs, under a 12-h light/12-h dark cycle (light phase: 20:00–8:00 h). Behavioural training and testing were conducted during the dark phase of the cycle. Food and water were available ad libitum throughout the experiment. All animal procedures were performed in accordance with UK Animals (Scientific Procedures) Act (1986) and associated guidelines. All efforts were made to minimise any suffering and the number of animals used. In these experiments, 2 groups of 10 animals were used. Group 1 was used for Experiments 1, 2a and 3; Group 2 was used for Experiment 2b. All statistical analyses used a significance level of 0.05.

### Apparatus

Objects were presented in an open-topped arena (90 × 100 cm^2^, walls 50 cm high) enclosed within a scaffold which supported a black cloth (150 cm high) so as to obscure external visual stimuli during experimentation. The arena floor was covered in sawdust. Behaviour was monitored and recorded for analysis using an overhead camera and DVD recorder. The stimuli were objects made from plastic ‘Duplo’ bricks (Lego UK Ltd, Slough, UK) which varied in colour and size (10 × 10 × 5 cm^3^ to 25 × 10 × 5 cm^3^) and which were secured to a concealed base to prevent them from being moved or tipped over. Multiple identical copies of each object were used so that different copies were presented in the sample and test phases, and each object was cleaned with absolute ethanol after each phase.

### General procedure

**Figure 1.**
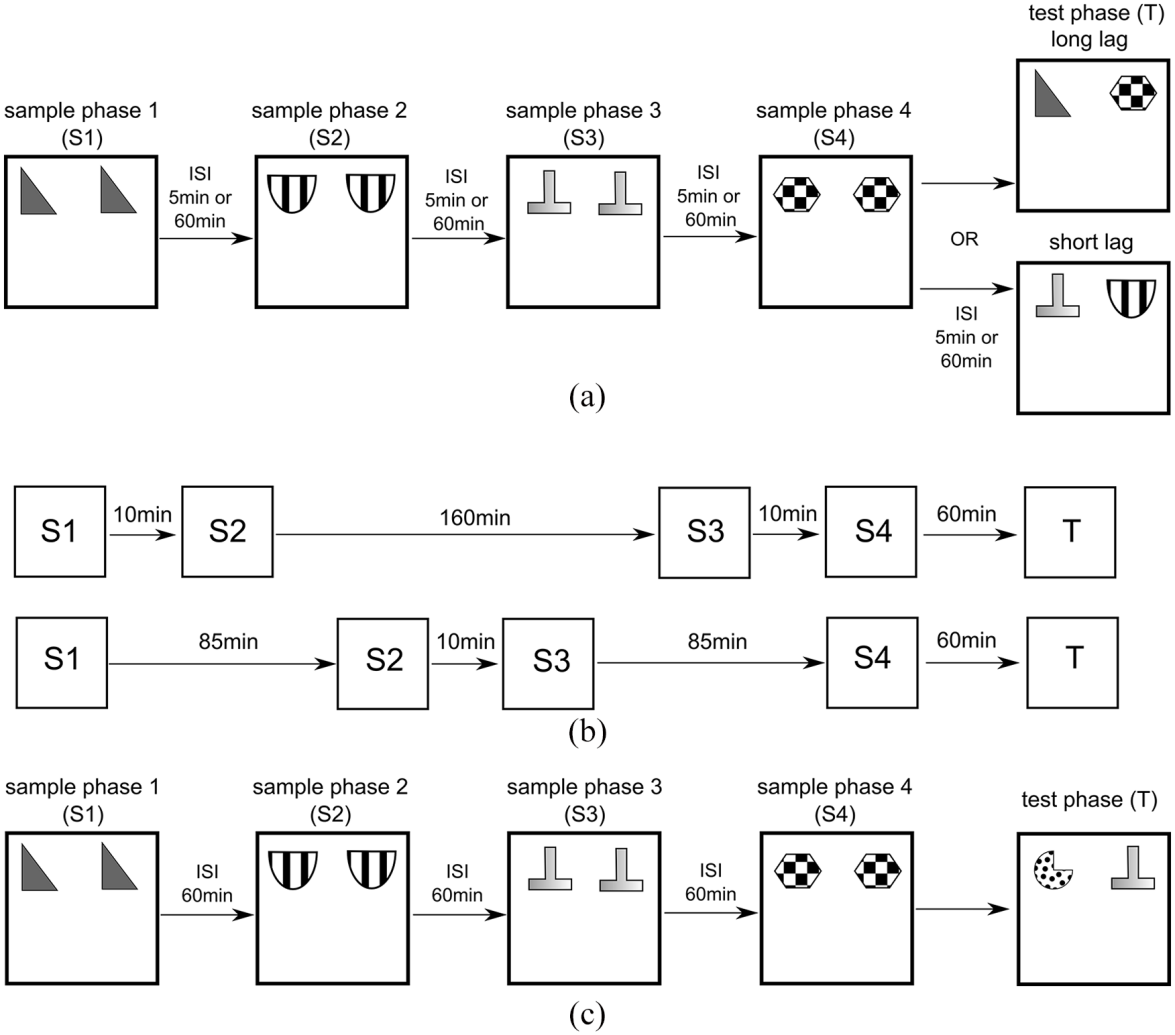
Variations of the four-object temporal order memory tasks tested. (a) Effect of different ISIs on temporal order memory performance. Animals were presented with a sequence of four objects with either a 5 min or 60 min ISI between each object. In the test phase, animals were presented with either the first and fourth object from the sequence (long lag) or the second and third object from the sequence (short lag). (b) Effect of varying the delay between S2 and S3 on temporal order memory performance. The delay between S1 and S4 was kept constant, while the delay between S2 and S3 was varied; in the short-delay condition, the delay between S2 and S3 was 10 min, and in the long-delay condition, the delay was 160 min. (c) Testing recognition memory of the objects presented in the sequence, the animals were presented with a sequence of four objects with an ISI of 60 min and the animals were presented with one object from the sequence and a novel object at the test phase.

All animals were habituated to the empty arena for 4 days prior to the start of behavioural testing. Each experiment comprised four sample phases (S1–S4) separated by an inter-sample interval (ISI). In each sample phase, the animals were presented with two copies of the sample object placed 15 cm from the side and back walls. The animals were allowed to explore the objects freely for 4 min and then removed from the arena and placed in the home cage for the ISI. Following the end of the ISI, the animals were placed back in the arena for the next sample phase and so on (Figure 1(a)). Different objects were presented in S1–S4, and following S4, the animal was placed back in the home cage for the duration of the retention delay, which was followed by a single test phase. Animals were exposed to one object sequence per week to minimise the interference between successive object sequences.

#### Experiments 1 and 2: testing order memory

To assess order memory, the animals were presented, in the test phase, with two sample phase objects, either the objects presented in the first (S1) and fourth (S4) sample phases (long lag) or the objects presented in the second (S2) and third (S3) sample phases (short lag) and were allowed to explore the objects for 3 min. In Experiment 1, the ISI was either 5 min or 60 min and the retention delay matched the ISI used between the sample phases. In Experiment 2, the ISI between S2 and S3 was either 10 min or 160 min. The interval between S1 and S4 was held constant at 180 min and the retention delay was 60 min (Figure 1(b)).

#### Experiment 3: testing familiarity discrimination

To assess familiarity discrimination, animals were presented, in the test phase, with a sample phase object and a novel object and were allowed to explore the objects for 3 min (Figure 1(c)). Intact recognition memory was demonstrated by a preference for exploring the novel object over the previously encountered object. The retention delay was 60 min. Across four separate sequences, each animal was tested for recognition memory of the object presented in each of the four sample phases. The order in which the different sample phases were tested was counterbalanced across animals.

### Data analysis and experimental design

#### Experimental design

A within-subject design was used to assess performance of the long-lag and short-lag discrimination within each ISI tested. For example, in Experiment 1, each animal performed the 5-min ISI task twice: memory for the long lag was tested in one run and memory for the short lag was tested in the other trial. The order in which the lags were tested was counterbalanced between animals. In Experiment 3, a within-subject design was used to test familiarity discrimination.

#### Object counterbalancing

To prevent exploratory behaviour being influenced by object preference, all animals were presented with the same two objects at the test phase irrespective of the lag to be tested. Within each lag condition, one of the objects was presented earlier in the sequence to half of the animals (in S1 or S2 as appropriate for the lag to be tested) and the second object was presented earlier in the sequence to the other half of the animals. The position of the temporally distant object occupied in the arena during the test phase was also counterbalanced within each run for each lag tested. In Experiment 3, all animals saw the same two objects in the test phase in each trial: one object had been presented in the sequence, that is, familiar and the other was novel. The object which acted as familiar and which acted as novel was counterbalanced across animals.

#### Analysis of exploration

Exploratory behaviour of an object was defined as the animal directing its nose towards the object at a distance of <2 cm. Other behaviours, for example, sitting on or resting against the object, were not scored.

Behavioural performance was assessed using a discrimination ratio which takes into account individual differences in the animals’ total object exploration during the test phase (Ennaceur and Delacour, 1988). To measure order memory, the time the animal spent exploring the object presented earlier in the sequence, that is, time old (*t_old_*), was compared to the time the animal spent exploring the object presented later in the sequence, that is, time recent (*t_recent_*), as a proportion of the total object exploration time using the following formula: (*t_old_* − *t_recent_*) / (*t_old_* + *t_recent_*). To measure recognition memory performance, the time the animal spent exploring the novel object, time novel (*t_novel_*), was compared to the time the animal spent exploring a familiar object, time familiar (*t_fam_*), as a proportion of the total object exploration time using the following formula: (*t_novel_* − *t_fam_*) / (*t_novel_* + *t_fam_*).

#### Analysis of discrimination across different delays

In Experiments 1 and 2a, discrimination in the short-lag condition across the different delays was assessed by plotting discrimination ratio against temporal ratio. The temporal ratio was calculated as previously reported in Hatakeyama et al. (2018), thus temporal ratio was calculated by the formula (Time S3 / Time S2), where Time S2 and Time S3 is the time between the end of either sample phase 2 or 3 and the start of the test phase. The discrimination ratio from the 60-min ISI condition in Experiment 1 was plotted against the temporal ratio as the overall length of the task matched that used in Experiment 2a.

#### Statistical analysis

Statistical comparisons between groups were made using either a one-way or multifactor analysis of variance (ANOVA) as appropriate; effect sizes eta, partial eta or generalised eta were calculated as appropriate for the analysis used (Lakens, 2013).

##### Experiment 1

A within-subject design was used to assess order memory (through discrimination of the old and recent object) in the short-lag (S2 versus S3) and long-lag (S1 versus S4) conditions. The effect of the different ISIs (5 or 60 min) was assessed in separate experimental sessions. Lag (short or long) was treated as the within-subject factor, and ISI (5 or 60 min) was treated as the between-subject factor.

##### Experiment 2

A within-subject design was used to assess order memory (through discrimination of the old and recent object), with a variable ISI between S2 and S3. Performance between the short and long lags was assessed in different sessions. Lag (short or long) was treated as the within-subject factor, and S2–S3 ISI (10 or 160 min) was treated as the between-subject factor.

##### Experiment 3

A four-way repeated-measures crossover design was used to assess familiarity discrimination between the objects presented in the sequence, compared to a novel object. Recognition of the objects presented in the different sample phases was assessed using a one-way ANOVA with object (from S1, S2, S3 or S4) as the within-subject factor. Post hoc test used a Bonferroni correction for multiple comparisons.

In all experiments, single-sample *t*-tests against 0 (two-tailed) were used to test whether animals in each condition showed significant discrimination between the old and recent object (Experiments 1 and 2) or between the familiar and novel object (Experiment 3).

#### Multiple linear regressions

The relationship between exploration across the four sample phases and discrimination of the order of object presentation was tested by multiple linear regression analyses. Before multiple linear regressions were performed, the data were tested to ensure it met seven assumptions. The seven assumptions were as follows: the data contained no outliers (standardised residuals lie between −3.29 and 3.29), collinearity (variance inflation factor (VIF) less than 10, tolerance greater than 0.1), independent errors (Durbin–Watson value greater than 1 but less than 3), random normally distributed errors (tested by examination of histogram of standardised residuals for normally distributed errors and examination of normal P-P plot of standardised residuals for linearity), homoscedasticity, linearity (tested by examination of the scatterplot of standardised predicted values) and non-zero variance (variance greater than 0).

We had no a priori prediction or evidence concerning which of the independent variables would be good predictors of discrimination of the order of object presentation; therefore, the multiple linear regression analysis was performed in a backward stepwise method. In the first stage of the analysis, all of the independent variables were entered into the model; if this failed to produce a model which predicted a significant amount of the variance in the discrimination performance, the variable with the least predictive value was removed and the regression analysis was repeated. This process was repeated until a model which predicted a significant amount of variance in the discrimination was identified or all variables were removed.

For the analysis of all experiments, the dependent variable was the discrimination ratio and the independent variables were exploration in sample phases 1–4. Additional independent variables used in each experiment were as follows: Experiment 1, lag and ISI; Experiment 2a, lag and delay between sample phases 2 and 3; Experiment 2b, delay between sample phases 2 and 3; and Experiment 3, position in sequence of object to be presented at the test phase.

#### Bayesian analysis

Bayesian *t*-tests were performed on the short-lag discrimination in Experiments 1, 2a and 2b to analyse the statistical strength of the observed null effects. Bayesian *t*-tests were performed using a Cauchy prior distribution and the hypothesis tested was that measure 1 ≠ measure 2, *Bayes*_10_ values were calculated as the weight of evidence in favour of *H*_0_ was tested and analyses were performed in JASP (JASP team, University of Amsterdam, v0.9.01).

*Bayes*_10_ values between 1.0 and 3.0 were interpreted as providing anecdotal evidence for *H*_0_. Values above 3.0 were interpreted as providing moderate evidence for *H*_0_, and values above 10.0 were interpreted as providing strong evidence for *H*_0_.

## Results

### Experiment 1: memory for order in a spontaneous preferential exploration task

**Figure 2.**
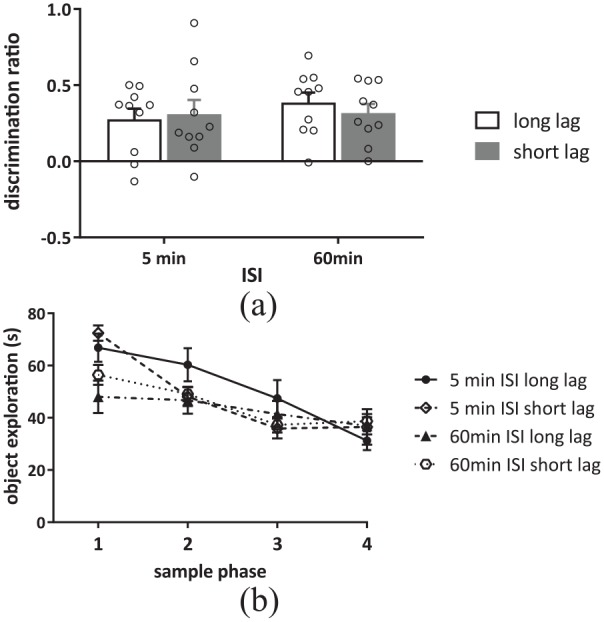
Performance in the temporal order memory task with an ISI of either 5 or 60 min. (a) Discrimination of the order of object presentation in the temporal order memory task with either a 5- or 60-min ISI in either the long-lag (S1 versus S4) or the short-lag (S2 versus S3) condition. (b) Exploration across the four sample phases in either the 5- or 60-min ISI condition. Data presented as (a) mean + SEM or (b) mean ± SEM, *n* = 10 for all.

As can be seen in Figure 2(a), all animals showed significantly greater exploration of the object presented earlier in the sequence irrespective of the ISI or the list position of the test objects (i.e. the lag between objects). Thus, two-way ANOVA with ISI and lag as factors revealed no significant interaction (*F*(1, 18) = 0.37, *p* = 0.55, $\eta_{G}^{2}=0.013$, $\eta_{p}^{2}=0.020$) and no significant main effect of either ISI (*F*(1, 18) = 0.89, *p* = 0.36, $\eta_{G}^{2}=0.017$, $\eta_{p}^{2}=0.047$) or lag (*F*(1, 18) = 0.04, *p* = 0.84, $\eta_{G}^{2}=0.002$, $\eta_{p}^{2}=0.002$). Further analysis confirmed that when the ISI was either 5 min or 1 h, animals showed a significant preference for the object presented earlier in the sequence in both the long-lag (5 min ISI: *t*(9) = 3.90, *p* = 0.004; 60 min ISI: *t*(9) = 9.09, *p* = 0.001) and the short-lag (5 min ISI: *t*(9) = 3.29, *p* = 0.009; 60 min ISI: *t*(9) = 7.59, *p* = 0.001) conditions.

#### Exploration in sample and test phases

There were changes in overall levels of object exploration in the sample phases, but not in the test phase. Statistical comparisons of the amount of exploration completed in each of the sample phases (see Figure 2(b)) using a three-way ANOVA with ISI, sample phase and lag as factors, revealed no significant three-way interaction (*F*(3, 54) = 1.01, *p* = 0.40, $\eta_{G}^{2}=0.0135$, $\eta_{p}^{2}=0.053$). There were significant two-way interactions between sample phase and lag (*F*(3, 54) = 3.35, *p* = 0.025, $\eta_{G}^{2}=0.0435$, $\eta_{p}^{2}=0.1570$) and sample phase and ISI (*F*(3, 54) = 4.95, *p* = 0.004, $\eta_{G}^{2}=0.2934$, $\eta_{p}^{2}=0.2156$) and a significant main effect of sample phase (*F*(3, 54) = 30.83, *p* = 0.001, $\eta_{G}^{2}=0.3204$, $\eta_{p}^{2}=0.6314$). Post hoc analysis of the significant main effect of sample phase revealed a significant decrease in exploration across the sample phases such that the exploration in S1 was significantly greater than in each of the subsequent sample phases (*p* < 0.001 for all) and exploration in S2 was significantly greater than the exploration completed in S3 (*p* = 0.09) and S4 (*p* = 0.002). Post hoc analyses further revealed that the significant interactions between sample phase and lag or ISI reflected different patterns of decreasing exploration across the sample phases in the different groups. Two-way ANOVA of exploration completed in the test phase with ISI and lag as factors revealed no significant interaction between ISI and lag (*F*(1, 18) = 1.1, *p* = 0.31, $\eta_{G}^{2}=0.0324$, $\eta_{p}^{2}=0.0580$) and no significant main effect of either ISI (*F*(1, 18) = 3.27, *p* = 0.09, $\eta_{G}^{2}=0.07645$, $\eta_{p}^{2}=0.1539$) or lag (*F*(1, 18) = 0.03, *p* = 0.86, $\eta_{G}^{2}=0.0010$, $\eta_{p}^{2}=0.0019$) (see Table 1 for mean).

**Table 1.** Total amount of object exploration completed in the test phase in each experiment.

Experiment	Condition	Exploration in test phase (s)		
		Long lag	Short lag	Old versus novel
1	5 min ISI	17.0 ± 2.3	19.5 ± 2.6	
	60 min ISI	24.6 ± 2.6	21.1 ± 2.6	
2	Long delay	30.4 ± 4.8	25.4 ± 2.2	
	Short delay	26.7 ± 3.6	26.8 ± 3.4	
2b	Long delay		25.7 ± 3.3	
	Short delay		36.5 ± 6.2	
3	S1 versus novel			27.7 ± 3.3
	S2 versus novel			26.6 ± 3.3
	S3 versus novel			29.9 ± 3.5
	S4 versus novel			31.7 ± 5.9

ISI: inter-sample interval; SEM: standard error of the mean.

Data presented as mean ± SEM.

### Experiment 2a: effects of varying the ISI between objects presented in a sequence

**Figure 3.**
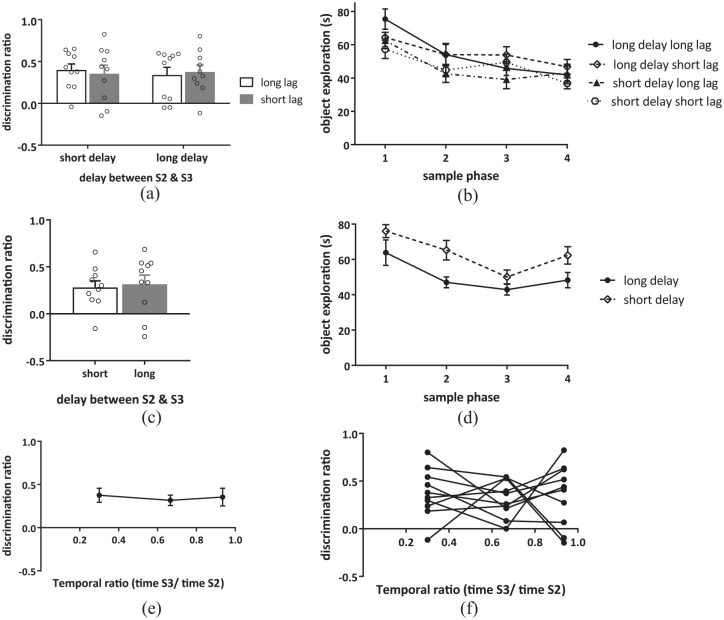
Performance in the temporal order memory task with a variable delay between S2 and S3. (a) Discrimination of the order of object presentation in the temporal order memory task for either the long lag or the short lag with either a long (160 min) or a short (10 min) delay between S2 and S3. (b) Exploration across the four sample phases of the temporal order memory task with either a long or a short delay between S2 and S3. (c) Discrimination of the order of object presentation in the temporal order task for a short lag (S2 versus S3) in a group of naïve animals with either a long or a short delay between S2 and S3. (d) Object exploration across the four sample phases of the temporal order memory task in the naïve group of animals with either a long or a short delay between S2 and S3. (e) Discrimination in the short-lag condition across the different temporal ratios tested, data from Experiment 1 and 2a are used. (f) Individual animal discrimination in the short-lag condition across the different temporal ratios tested. Data presented as (a, c) mean + SEM or (b, d, e) mean ± SEM, *n* = 10 for all.

In this experiment, the ISI between S2 and S3 was varied, while the time interval between S1 and S4 remained at 180 min. The results show that varying the ISI had no effect on the animals’ ability to discriminate between the S2 and S3 objects or between S1 and S4 objects (see Figure 3(a)). Two-way ANOVA with ISI and lag as factors revealed no significant interaction (*F*(1, 18) = 0.19, *p* = 0.67, $\eta_{G}^{2}=0.006$, $\eta_{p}^{2}=0.011$) and no significant main effect of lag (*F*(1, 18) = 0.01, *p* = 0.97, $\eta_{G}^{2}=0.00006$, $\eta_{p}^{2}=0.0001$) or ISI (*F*(1, 18) = 0.06, *p* = 0.82, $\eta_{G}^{2}=0.001$, $\eta_{p}^{2}=0.003$). Additional analysis confirmed that in both delay conditions, the animals showed significant discrimination between the old and recent object in both the long-lag (10 min ISI: *t*(9) = 5.59, *p* = 0.001; 160 min ISI: *t*(9) = 3.70, *p* = 0.005) and the short-lag (10 min ISI: *t*(9) = 3.45, *p* = 0.007; 160 min ISI: *t*(9) = 4.63, *p* = 0.001) conditions.

#### Exploration in sample and test phases

There were changes in overall levels of object exploration in the sample phases, but not in the test phase. Analysis of the amount of exploration completed across the sample phases in the different ISI conditions revealed no significant interaction between sample phase, ISI and lag (*F*(3, 54) = 0.93, *p* = 0.43, $\eta_{G}^{2}=0.0114$, $\eta_{p}^{2}=0.0491$). There was a significant interaction between sample phase and lag (*F*(3, 54) = 3.43, *p* = 0.02, $\eta_{G}^{2}=0.0409$, $\eta_{p}^{2}=0.1560$) and a significant main effect of sample phase (*F*(3, 54) = 13.26, *p* = 0.001, $\eta_{G}^{2}=0.2455$, $\eta_{p}^{2}=0.4243$) and ISI (*F*(1, 18) = 5.22, *p* = 0.04, $\eta_{G}^{2}=0.0604$, $\eta_{p}^{2}=0.2248$) (see Figure 3(b)). Analysis of the amount of exploration completed in the test phase revealed no significant interaction between ISI and lag (*F*(1, 18) = 0.40, *p* = 0.54, $\eta_{G}^{2}=0.0124$, $\eta_{p}^{2}=0.0216$) and no significant main effect of either ISI (*F*(1, 18) = 0.11, *p* = 0.74, $\eta_{G}^{2}=0.00271$, $\eta_{p}^{2}=0.0062$) or lag (*F*(1, 18) = 0.36, *p* = 0.56, $\eta_{G}^{2}=0.0112$, $\eta_{p}^{2}=0.0196$) (see Table 1 for mean).

### Experiment 2b: effects of varying the ISI between objects presented in a sequence in naïve animals

Experiment 2a revealed that the ISI between S2 and S3 did not impact the animals’ ability to discriminate the order of object presentation. As the animals used in Experiment 2a had some prior experience of the task from Experiment 1, Experiment 2a was repeated with a group of naïve animals.

Here, in both ISI conditions, the animals showed significant discrimination between the old and recent object (10 min ISI: *t*(9) = 4.02, *p* = 0.003; 160 min ISI: *t*(9) = 3.22, *p* = 0.01), and ANOVA revealed no significant main effect of ISI (*F*(1, 9) = 0.09, *p* = 0.77, $\eta_{G}^{2}=0.042$, $\eta_{p}^{2}=0.095$, *ω*^2^= −0.002) (see Figure 3(c)).

These results confirm that varying the ISI between S2 and S3 does not influence the animals’ ability to discriminate object order.

#### Exploration in sample and test phases

There were changes in overall levels of object exploration in the sample phases, but not in the test phase. Analysis of the amount of exploration completed in the sample phases revealed no significant interaction between ISI and sample phase (*F*(3, 27) = 0.51, *p* = 0.68, $\eta_{G}^{2}=0.0243$, $\eta_{p}^{2}=0.0540$) although there was a significant main effect of ISI (*F*(1, 9) = 14.73, *p* = 0.004, $\eta_{G}^{2}=0.2103$, $\eta_{p}^{2}=0.6208$) and sample phase (*F*(3, 27) = 10.95, *p* = 0.001, $\eta_{G}^{2}=0.3097$, $\eta_{p}^{2}=0.5489$) (see Figure 3(d)). The significant effect of ISI reflected the higher level of exploration completed across all the sample phases in the 10-min ISI condition. Post hoc analysis of the main effect of sample phase revealed that in both ISI conditions, the animals spent significantly less time exploring the object presented in S3 than the object presented in S1 (*p* = 0.002) or S2 (*p* = 0.03). Analysis of the amount of exploration completed in the test phase revealed no significant main effect of ISI (*F*(1, 9) = 1.92, *p* = 0.20, $\eta_{G}^{2}=0.1988$, $\eta_{p}^{2}=0.8856$, *ω*^2^ = 0.0876) (see Table 1 for mean).

### Discrimination of order of object presentation across temporal ratios

When discrimination in the short-lag condition was plotted against temporal ratio (see Figure 3(e) and (f)), it was found that discrimination did not significantly change as the delay between the object presentations changed; one-way ANOVA analysis revealed no significant main effect of temporal ratio (*F*(2, 27) = 0.20, *p* = 0.82, $\eta_{p}^{2}=0.0073$).

### Bayesian analysis of temporal order discrimination in Experiments 1 and 2

Manipulation of the ISI in Experiments 1 and 2 did not significantly alter the animals’ ability to discriminate the order of object presentation. While the frequentist measures used to examine the data provided clear evidence to reject the alternative hypothesis, that is, that changes in delay altered performance, they did not provide an assessment of the strength of evidence to support *H*_0_. Thus, we performed Bayesian *t*-test analyses on the discrimination ratios of the short-lag condition in Experiments 1 and 2.

**Figure 4.**
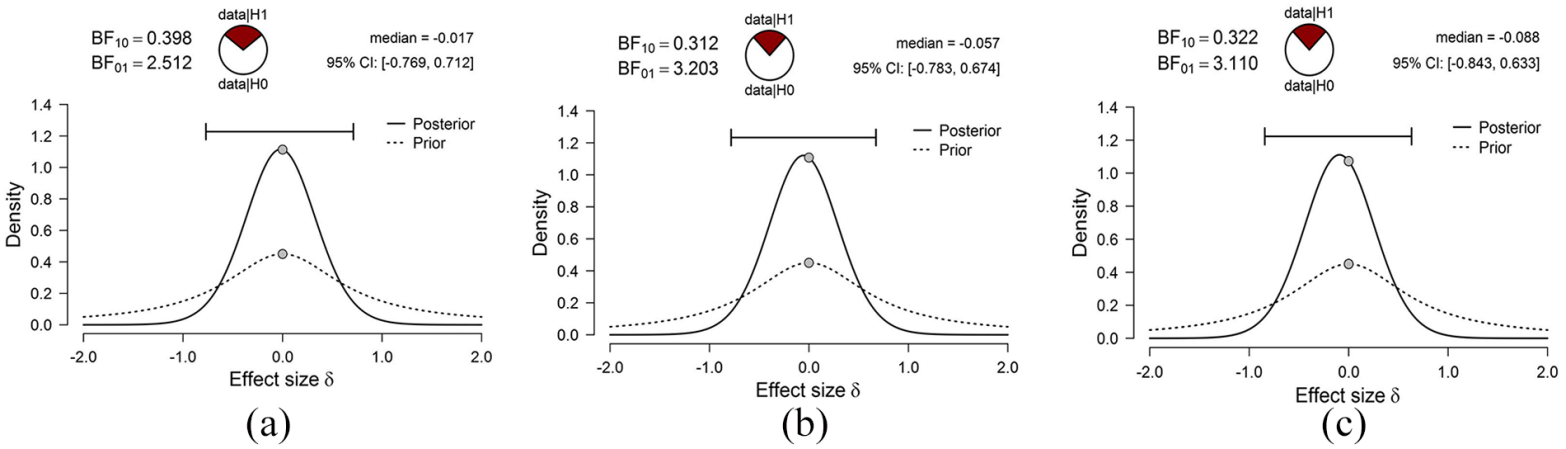
Prior and posterior distributions for the Bayesian *t*-tests performed on the short-lag discrimination in (a) Experiment 1, (b) Experiment 2a and (c) Experiment 2b. Figures from JASP.

A Bayesian *t*-test of discrimination in the short-lag condition between the 5- and 60-min ISI in Experiment 1 revealed anecdotal evidence in support of the *H*_0_ (*BF*_10_ = 2.51). Analysis of performance in the short-lag discrimination between the long (160 min) and short (10 min) delays in Experiments 2a and 2b again found moderate evidence in support of *H*_0_ (Experiment 2a: *BF*_10_ = 3.20; Experiment 2b: *BF*_10_ = 3.11) (see Figure 4 for prior and posterior distributions).

### Experiment 3: memory for order is not a function of object familiarity

**Figure 5.**
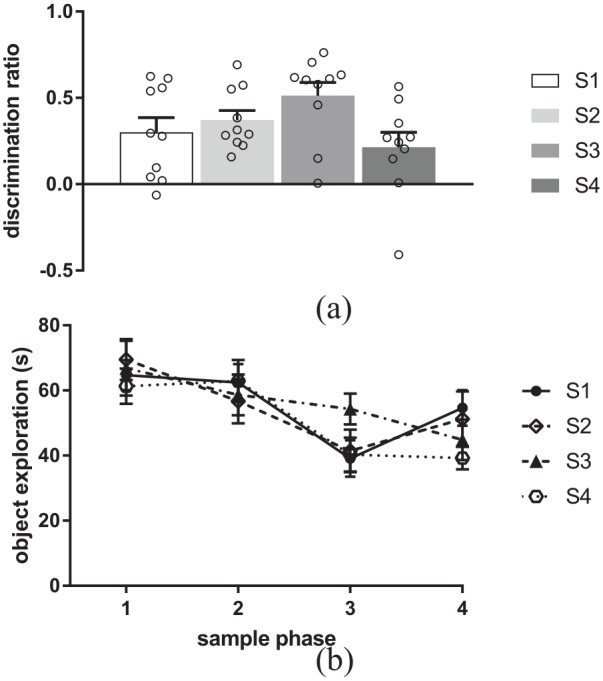
Recognition of the objects presented in the temporal order memory task. (a) Discrimination between a novel object and each of the objects presented in the four-object sequence. (b) Exploration across the four sample phases in the temporal order memory task. Data presented as mean + SEM, *n* = 10.

It has been argued that discrimination of old from recent objects can be achieved solely using object familiarity (Ennaceur, 2010), as more time has passed since the old object was experienced it will be less familiar, so this issue was examined in Experiment 3. Figure 5(a) shows no difference in the level of discrimination between the objects presented in the sequence (S1, S2, S3, S4) and a novel object (*F*(3, 27) = 2.23, *p* = 0.11, $\eta_{G}^{2}=0.183$, $\eta_{p}^{2}=0.199$, *ω*^2^ = 0.100). Additional analysis confirmed significant discrimination between each of the objects presented and a novel object (S1: *t*(9) = 3.55, *p* = 0.006; S2: *t*(9) = 6.69, *p* = 0.001; S3: *t*(9) = 6.62, *p* = 0.001; S4: *t*(9) = 2.51, *p* = 0.03).

#### Exploration in sample and test phases

The amount of object exploration completed within each sample phase is shown in Figure 5(b). ANOVA with sample phase and object as factors revealed no significant interaction between sample phase and object (*F*(9, 81) = 1.65, *p* = 0.12, $\eta_{G}^{2}=0.0513$, $\eta_{p}^{2}=0.1548$) and no significant main effect of object (*F*(3, 27) = 0.31, *p* = 0.82, $\eta_{G}^{2}=0.0115$, $\eta_{p}^{2}=0.0330$); however, there was a significant main effect of sample phase (*F*(3, 27) = 28.85, *p* = 0.001, $\eta_{G}^{2}=0.1918$, $\eta_{p}^{2}=0.7622$). Post hoc analysis revealed that across all conditions, the animals completed significantly less exploration in sample phases 3 and 4 compared to sample phases 1 and 2 (S1 versus S3: *p* = 0.001; S1 versus S4: *p* = 0.002; S2 versus S3: *p* = 0.001; S2 versus S4: *p* = 0.001). Analysis of the amount of exploration completed in the test phase revealed no significant main effect of object (*F*(3, 27) = 0.27, *p* = 0.84, $\eta_{G}^{2}=0.0236$, $\eta_{p}^{2}=0.0295$, *ω*^2^ = −0.0611) (see Table 1 for mean).

### Relationship between exploration in sample phases and discrimination of the order for object presentation

Multiple linear regression analyses were used to investigate the relationship between object exploration in the sample phases and discrimination performance. Initial analysis revealed that the data from each experiment met the seven assumptions required for this analysis. Analysis of the standard residuals revealed the data contained no outliers (standardised residual min. = −2.18, standardised residual max. = 2.07). Tests of collinearity indicated that multicollinearity was not a concern (tolerance = 0.41–0.95, VIF = 1.05–2.46). The data met the assumption of independent errors (Durbin–Watson value = 2.01–2.36). Inspection of the histogram of standardised residuals indicated that the data contained approximately normally distributed errors, as did the normal P-P plot of standardised residuals, which showed points that were not completely on the line, but close. The scatterplot of standardised predicted values showed that the data met the assumptions of homogeneity of variance and linearity and also met the assumption of non-zero variances (all variances > 0).

In Experiment 1 using a stepwise backward multiple linear regression method analysis, a model with exploration in sample phases 1–4, lag and ISI as independent variables did not explain a significant amount of the variance in the discrimination performance in the test phase (*F*(6, 33) = 0.955, *p* = 0.471, *R*^2^ = 0.15, $R_{Adjusted}^{2}=-0.01$); furthermore, none of the individual variables significantly predicted discrimination performance (lag beta = −0.15, *t*(33) = −0.81, *p* = 0.43; ISI beta = −0.17, *t*(33) = −0.87, *p* = 0.39; exploration in S1 beta = 0.22, *t*(33) = 0.89, *p* = 0.38; exploration in S2 beta = −0.42, *t*(33) = −1.91, *p* = 0.07; exploration in S3 Beta = −0.06, *t*(33) = −0.30, *p* = 0.77; exploration in S4 beta = −0.12, *t*(33) = −0.68, *p* = 0.50). Stepwise backward analyses identified a model in which exploration in sample phase 2 predicted a significant amount of the variance in discrimination (*F*(1, 38) = 4.23, *p* = 0.047, *R*^2^ = 0.10, $R_{Adjusted}^{2}=0.08$) as well as significantly predicting discrimination performance (beta = −0.32, *t*(38) = −2.06, *p* = 0.047). Indeed, there was a negative correlation between exploration in sample phase 2 and discrimination (Pearson’s *r* = −0.32, *p* = 0.02). Thus, lower levels of object exploration in sample phase 2 resulted in greater discrimination of the order of object presentation; however, this relationship was independent of the lag or ISI tested.

In Experiment 2a, a stepwise backward multiple linear regression analysis with lag, delay between sample phases 2 and 3 and exploration in sample phases 1–4 as independent variables found that the model did not explain a significant amount of the variance in discrimination (*F*(6, 33) = 1.74, *p* = 0.14, *R*^2^ = 0.24, $R_{Adjusted}^{2}=0.10$) and none of the individual variables significantly predicted discrimination performance (lag beta = −0.01, *t*(33) = −0.01, *p* = 0.99; delay between S2 and S3 beta = 0.01, *t*(33) = 0.02, *p* = 0.98; exploration in S1 beta = −0.28, *t*(33) = −1.59, *p* = 0.12; exploration in S2 beta = 0.34, *t*(33) = 2.03, *p* = 0.05; exploration in S3 beta = −0.30, *t*(33) = −1.73, *p* = 0.09; exploration in S4 beta = −0.03, *t*(33) = −0.17, *p* = 0.87). Subsequent stepwise backward analyses produced a model in which exploration in sample phases 1, 2 and 3 as independent variables predicted a significant amount of the variation in discrimination (*F*(3, 36) = 3.77, *p* = 0.02, *R*^2^ = 0.24, $R_{Adjusted}^{2}=0.18$), and in this model, exploration in sample phase 2 also significantly predicted discrimination performance in a positive direction (beta = 0.34, *t*(36) = 2.23, *p* = 0.03) but exploration in sample phases 1 and 3 did not (sample phase 1 beta = −0.28, *t*(36) = −1.87, *p* = 0.07; sample phase 3 beta = −0.30, *t*(36) = −1.95, *p* = 0.06).

In Experiment 2b, a stepwise backward multiple linear regression with delay between sample phases 2 and 3 and exploration in sample phases 1–4 as independent variables found that the model did not predict a significant amount of the variance in the discrimination performance (*F*(5, 14) = 0.29, *p* = 0.91, *R*^2^ = 0.09, $R_{Adjusted}^{2}=-0.23$) and none of the individual variables significantly predicted discrimination performance (delay between sample phase 2 and sample phase 3 beta = 0.04, *t*(14) = 0.12, *p* = 0.91; exploration in S1 beta = −0.32, *t*(14) = 1.13, *p* = 0.28; exploration in S2 beta = 0.09, *t*(14) = 0.25, *p* = 0.81; exploration in S3 beta = 0.05, *t*(14) = 0.14, *p* = 0.89; exploration in S4 beta = 0.03, *t*(14) = 1.0, *p* = 0.93). Subsequent backward analyses did not identify a model which significantly predict the variation in the discrimination of order of object presentation (model 5, independent variable exploration in sample phase 1 *F*(1, 18) = 1.58, *p* = 0.23, *R*^2^ = 0.08, $R_{Adjusted}^{2}=0.03$). Thus, in Experiment 2b, there was no statistically significant relationship between exploration in the sample phases and discrimination of the order of object presentation.

In Experiment 3, a stepwise backward multiple linear regression with object to be discriminated and exploration in sample phases 1–4 as independent variables found that the model did not predict a significant amount of the variance in the discrimination of the novel object (*F*(5, 34) = 1.84, *p* = 0.13, *R*^2^ = 0.21, $R_{Adjusted}^{2}=0.10$) and none of the variables significantly predicted discrimination performance (object tested beta = −0.16, *t*(34) = −0.99, *p* = 0.33; exploration in S1 beta = 0.17, *t*(34) = 0.86, *p* = 0.40; exploration in sample phase 2 beta = 0.19, *t*(34) = 0.95, *p* = 0.35; exploration in S3 beta = 0.32, *t*(34) = 1.73, *p* = 0.09; exploration in S4 beta = −0.29, *t*(34) = 0.32, *p* = 0.15). Subsequent stepwise backward analyses resulted in a model where exploration in sample phase 3 as an independent variable significantly predicted the variance in the discrimination of the novel object (*F*(1, 38) = 5.80, *p* = 0.02, *R*^2^ = 0.13, $R_{Adjusted}^{2}=0.11$) and predicted discrimination performance (beta = 0.36, *t*(38) = 2.41, *p* = 0.02). The significant relationship between exploration in sample phase 3 and discrimination of the novel object reflected a positive correlation between the two variables (Pearson’s *r* = 0.36, *p* = 0.01) although this relationship was independent of which object was to be discriminated during the test phase.

## Discussion

A range of cognitive mechanisms have been proposed which could underlie an animal’s ability to judge the order of items in a sequence (for review see Marshuetz and Smith, 2006), one of which is the trace-decay hypothesis (Staddon and Higa, 1999) in which discrimination of the order of object presentation increases as the temporal separation between object presentations increases. Here, increasing the ISI between object presentations did not change discrimination performance in either a long-lag or short-lag condition and importantly discrimination was not significantly different between the short-lag and the long-lag conditions. In addition, manipulating the ISI between S2 and S3 to create either a short (10 min) or very long (160 min) ISI did not alter performance. Finally, recognition memory of the four objects presented in the sequence was not found to be significantly different.

To understand the significance of the lack of effect of manipulating the ISI, we performed Bayesian *t*-tests to measure the strength of evidence for *H*_0_. The Bayes factors of 2.5–3.25 obtained in the analyses indicate that the data are two and a half to three times more likely to be explained by *H*_0_ than the alternative hypothesis. While these levels are not considered strong evidence in support of *H*_0_, a significantly larger number of animals would have had to have been tested to produce this level of evidence. In addition, while this study employed a range of delays we did not test either very short (seconds or minutes) or longer delays (24 h or longer); therefore, we cannot rule out the possibility that different effects or different mechanisms may underlie temporal judgements on different timescales.

Levels of object exploration decreased across the sample phases which could influence encoding and subsequent object memory. Therefore, the relationship between sample phase exploration and test phase discrimination performance was examined using multiple linear regression analyses. In Experiments 1, 2a and 3, but not in Experiment 2b, a significant relationship between sample phase exploration and discrimination was found; however, there was not a consistent model identified in each experiment. Experiment 1 found a significant negative relationship between S2 exploration and discrimination, while in Experiment 2a, this relationship was in a positive direction. Furthermore, the models predicted a relatively low amount of the variance in discrimination performance (Experiment 1, 8%; Experiment 2a, 18%), hence it is not clear what role any relationship might play. Future studies could examine the effects of systematically manipulating sample exploration levels on performance.

The insensitivity of temporal order judgements to delay has previously been reported, both in non-human primates (Templer and Hampton, 2013) and in rodents, albeit in a task in which the delay between the second sample phase and test phase was varied up to 24 h (Mitchell and Laiacona, 1998). Other studies, using two-object or five-object versions, have shown weaker discrimination when delays were shorter (Hatakeyama et al., 2018; Tam et al., 2014). In this study and in that of Mitchell and Laiacona (1998), the animals were tested during the dark phase of the animals’ daily cycle, whereas Tam et al. (2014) and Hatakeyama et al. (2018) tested temporal order memory during the light phase. Thus, possible explanation for the inconsistencies in the results could be in these methodological differences.

Previous studies have also reported ‘temporal separation effects’ (e.g. Fortin et al., 2002; Kesner et al., 2002; Templer and Hampton, 2013), that is, discrimination of the item order improved as the number of intervening items between the to-be-discriminated stimuli increased. However, here discrimination in the long-lag condition (two intervening stimuli) was not significantly different from the short-lag condition (no intervening stimuli), thus no ‘temporal separation effect’ was observed. In studies which report a clear temporal separation effect, the ISIs are typically less than 5 min, whereas in this study, the ISI was either 5 min or 1 h. With longer intervals between items, each item may be more discriminable as it is represented in memory along with its own temporal context distinct from that of the other stimuli (Howard and Kahana, 2002; Polyn et al., 2009). Indeed, theories of temporal order memory argue that time itself is an important contextual stimulus which changes over the duration of the testing period, thus dissimilar temporal cues serve to disambiguate the order of stimuli (Manns et al., 2007). In addition, the longer ISI in the present design meant that there was no contiguity effect, that is, it is unlikely that items presented close together in time become associated with one another (Kahana, 1996). Therefore, discrimination between the temporally adjacent items (short-lag condition) in this study was achieved just as well as between temporally distant items (long-lag condition) because the temporal cues under both conditions were sufficiently distinct.

The finding that rats show a preference for an earlier list item without training or reward is important, as it has been argued that spontaneous memory tasks more closely model human episodic learning and memory (Ennaceur, 2010). An important question, however, concerns the nature of the cognitive process underlying such spontaneous preference. The preferential exploration of the earlier list items was shown not to be due to these items being forgotten and therefore regarded as ‘novel’, as Experiment 3 showed that recognition memory for the list items was intact. The absence of a temporal separation effect and the demonstration that discrimination between a test item and a novel object did not vary as a function of the test items position in the list clearly show that the animals are making order judgements, at least in part, independent of familiarity discrimination processes. This conclusion is supported by findings from other studies which have shown that hippocampal lesions disrupt memory for order without disrupting familiarity discrimination, which suggest that these two processes are mediated by different cellular mechanisms (Fortin et al., 2002).

This study was able to rule out trace decay or familiarity processes as key to order memory, and in addition, previous accounts of temporal order memory (Tam et al., 2014) have explained performance using a model of stimulus learning proposed by Wagner and colleagues (Brandon et al., 2003; Wagner, 1981) which again our results do not support. In the Wagner model presentation of a stimulus, such as an object, results in elements of that object representation entering a primary state of activation (A1) and then to a decayed state of activation (A2) before becoming inactive. A stimulus will only produce a response, such as exploration, if the representation is active and greater exploration is generated if the stimulus is in the A1 state compared to A2. Using this theory, in the temporal order task, as all of the objects have been presented at different times, the elements of the objects will exist in different A2 states, and thus generate different levels of exploration. Further increasing the delay between stimuli should increase the difference in the level of A2 activity and change discrimination performance, which is not what was observed in this study.

Understanding how animals remember order information is critical to understanding the nature of episodic-like memory and its relationship to episodic memory in humans. While we have provided evidence that temporal decay of the object memory is not critical for the judgement of order, we cannot exclude other mechanisms such as judgements based on monitoring how long ago an item was encountered (Roberts et al., 2008), temporal context (Mann et al., 09) or item–item associations (Marshuetz and Smith, 2006). There are alternative mechanisms which may have altered the memory strength of the objects which are independent of time. One possible alternative explanation is provided by the cognitive event model (Radvansky et al., 2010, 2011) which has proposed that event boundaries, such as moving between rooms in humans or the animals entering and leaving the arena in this study, can act as event boundaries which affect how items are remembered. In this study, each sample phase could have altered the memory strength of the previous event in a time independent manner, thus creating differences in memory strength which could support the observed performance of order discriminations.

Here, naïve animals were able to discriminate order of object presentation with the short ISI between S2 and S3 when they had no prior knowledge that these objects would be presented again, thus animals can encode the information concerning the position of an item in a list or sequence without extensive or reinforced training protocols and using trial unique stimuli; hence, familiarity judgements are not the primary mechanism which underlies the expression of temporal order memory. Concerning the neurobiological mechanisms underlying order memory, the hippocampus and medial prefrontal cortex (mPFC) have both been implicated in temporal order memory (DeVito and Eichenbaum, 2011). Specifically, changes in firing patterns of neurons in the hippocampus across delay periods may, as an ensemble, underpin order memory and representations of temporal context (MacDonald et al., 2011; Manns et al., 2007). Furthermore, our results using a two-item temporal order memory task have shown that such memory is subserved by a direct hippocampal CA1-mPFC interaction (Barker and Warburton, 2011; Barker et al., 2017). Clearly, further work using paradigms, such as the one presented here, is required to fully differentiate the contributions of these brain regions to memory for order.
